# DATA 5.0—Data Acquisition, Translation & Analysis—a prospective urooncological data warehouse for the 21st century

**DOI:** 10.3389/fdgth.2025.1530321

**Published:** 2025-03-27

**Authors:** Viktoria Schütz, Christine Geisler, Mathias Rath, Sarah Böning, Thomas Treber, Albrecht Stenzinger, Alexander Brobeil, Oliver Reinhard, Anette Duensing, Stefan Duensing, Markus Hohenfellner, Magdalena Görtz

**Affiliations:** ^1^ Department of Urology, University Hospital Heidelberg, Heidelberg, Germany; ^2^ Molecular Urooncology, Department of Urology, University Hospital Heidelberg, Heidelberg, Germany; ^3^ Center for Digitalization and Information Technology, University Hospital Heidelberg, Heidelberg, Germany; ^4^ Institute of Pathology, University Hospital Heidelberg, Heidelberg, Germany; ^5^ Precision Oncology of Urological Malignancies, Department of Urology, University Hospital Heidelberg, Heidelberg, Germany

**Keywords:** medical database, tumor database, urological tumors, data warehouse, big data analysis, individualized cancer treatment, prospective data acquisition, biobanks

## Abstract

**Background:**

Prospective data registration is the basis of clinical oncological research. Commonly, case documentation is restricted to studies investigating a defined hypothesis. Only few institutions prospectively register all oncological patients with a reliable, sustainable and continuous follow-up infrastructure. The Department of Urology of the Heidelberg University Hospital started its prospective tumor data base in 1992. Since then, the clinical course of all oncological in-patients is continuously registered within a life-long follow-up (success rate: 93%). Associated tumor tissue is stored in the Heidelberg Biobank. In 2005, the transfer of this invaluable registry from the initial InterSystemsCache®/KRAZTUR system to a modern data warehouse was initiated. However, the transfer of existing data into a new environment proved to be technically challenging.

**Objective:**

To migrate the existing data into a modern data warehouse (DATA 5.0) while maintaining data extraction functions. Additional requirements included FHIR connectivity, big data analyses and AI applications.

**Methods:**

Together with SAP SE, DATA 5.0 was developed. Based on SAP HANA® (High Performance Analytic Appliance) it allows data registration and analysis with third party analytical tools. The project was supported by members of the SAP SE executive board and funded by the Dietmar Hopp Foundation.

**Results:**

Data Acquisition, Translation & Analysis 5.0 (DATA 5.0), a web-based tool for data registration, preservation and analysis of treatment and follow-up data, was developed to proof-of-concept stage. DATA 5.0 was then implemented into clinical practice replacing the previous system. As of today, 15,345 oncological patients and 6.7 Mio. data points are registered.

**Conclusion:**

Prospective long-term data was successfully migrated into DATA 5.0, allowing data preservation, flexibility and capabilities for future data sources. DATA 5.0, together with associated tumor tissue, is a lighthouse platform for oncological research, with capability for third party analytical tools, big data analysis and AI applications including training of digital twin models.

## 1 Introduction

The systematic collection of data from cancer patients together with tissue and liquid biobanking is pivotal for oncological research (1–5). Patient data are needed for virtually every aspect of biomarker development, patient stratification, clinical trial development and implementation, as well as the implementation of novel therapies. In addition, various aspects of basic research rely on patient data and tissue samples. A well-established database with the collection of longitudinal, high quality and curated data allows research and clinical practice to go hand in hand to constantly improve patient care (6). The concept of collecting and storing data of cancer patients has had significant momentum over the past decades. A tumor data bank provides comprehensive storage of different kind of information, from basic patient characteristics (e.g., age, medical history), tumor characteristics (e.g., location, grade group, TNM classification), imaging and treatment data. It could also contain information from clinical trials and research. Tumor data banks can be found at hospitals, health care and research institutions, for specific tumor entities, as part of specific trials, but also as national registries (7–10). Examples for web-based documentation systems for collecting patient data have been established in Sweden (11) and the United States (12). At the Department of Urology at Heidelberg University Hospital the prospective collection of data from patients with urological tumors began in the early 1990s (1).

This expansion of efforts to collect data of cancer patients is also a reflection of the fact that the data available for each individual cancer patient has increased exponentially over the past decade (13). In general, these include primary data, such as age, gender, underlying medical conditions, risk factors, lab results, BMI, family history, imaging, pathology, survival data, therapy responses, outcome data, treatment/surgery, adverse effects. Information from genetic analysis and molecular data is considered secondary data. While data collected from research (e.g., molecular research, biomarker studies) is regarded as tertiary data, which in the future should also be part of medical databases. Another important aspect is time. Patient survival data needs to be collected and stored over long time intervals, which, for example for prostate or renal cancer, may comprise over two decades. To ensure data quality is crucial. This involves identification of adequate data sources and thorough control of all the data added into the databank in a standardized manner by highly trained staff. This can be achieved by adequate training of staff, limiting access to the databank, constant data validation and data integrity.

In the late 1980s and early 1990s a prospective data base of patients with urological tumors was implemented at the Department of Urology of the University Hospital Heidelberg. This tumor documentation system includes all relevant primary data. Most importantly, all patients are followed up regularly by a team of highly trained medical documentalists. High quality survival data are collected over a long time frame for research studies through a structured patient after-care program. Patients are regularly contacted and reminded of their next follow-up appointment, which is very well received by patients and their treating urologists (1). By 2022 the database consisted of approx. 14,200 patients with over 137,000 surveys and approx. 3.6 million data points.

Storing, preserving, and retrieving patient data is crucial for research and personalized medicine. Especially real-world data and evidence can be crucial in precision medicine (14). Over the past decades many scientific publications have been generated using information retrieved from the tumor documentation system (1, 15–17). The value added by associating patient information with tissue samples for cancer research is immense. Tissue samples at University Hospital Heidelberg are stored in the NCT Biobank, which is part of the German Biobank Alliance (18) and one of the first European research tissue banks accredited according to ISO/IEC 17020 (19). Different biobanks and biobank collaborations have been established across Europe (20). However, the value of data received from the analysis of tissue samples, can be immensely increased by adding clinical and long-term survival information (21).

Even in healthcare the value of different kinds of data from various sources has not only been recognized for its impact on research, but has also been regarded in an economic sense. Therefore, all data needs to be stored in a manner that will be available and retrievable in the future. The database created in Heidelberg in the 1990s was based on an InterSystemsCache®/KRAZTUR data base developed by Ellsässer et al. (22, 23). However, after over two decades this system had become outdated and the need for a new, modern, extendable and non-proprietary data warehouse arose. Over fifteen years ago the search for a new solution to preserve all valuable previously collected data began. With the need to preserve all previous data and make it available for future research, a new cloud-based data warehouse (DATA 5.0—Data Acquisition, Translation and Analysis) was eventually developed together with experts from different fields (medical users, researchers, documentation staff, software developers, hospital IT service). Besides technical aspects, ethical and data protection standards are highly important. Key ethical aspects include information on the type of data stored with patients having a right to oppose and having information deleted in case of consent withdrawal. Patients need to give written, informed consent to participate (24). By complying with ethical standards and data protection regulations, acceptance of such a data base is fostered. The new data-warehouse should not only store and preserve existing data but should also allow the addition of new data including information from imaging, pathological results, as well as genomic analysis. At the same time the platform should be accessible for big data analytical tools and connect to device integration platforms such as OP 4.1 (25).

## 2 Methods

### 2.1 Workflow of planning a new data warehouse

The initial scoping started after being initiated by then SAP SE board member Bernd Leukert in 2018 together with an expert team of SAP SE lead by Dr. Anette Großmüller. During the first meetings the challenge of preserving all data registered in the outdated tumor documentation system InterSystemsCache®/KRAZTUR was discussed. Also, the objectives and requirements for a new database were highlighted. Besides storing previous data and making it accessible for future research and big-data analysis, the new database should allow the addition of more information (e.g., imaging data, molecular and genetic data), i.e., identification of current and future data sourced and formats. A data standard was chosen so that in the future IT will be able to communicate in a standardized manner across institution boundaries, making data accessible and available online. FHIR® has many advantages over other standards. The FHIR® standard (Fast Healthcare Interoperable Resources, pronounced “fire”) was created by Health Level Seven International (HL7) (26). The standard supports the exchange of data between software systems in the healthcare sector and combines the advantages of established standard product lines with those of current web standards (XML, JSON, ATOM, HTTPS, OAuth, etc.) and places a focus on ease of implementation (26, 27). One of the main objectives in using FHIR® was a simple and fast implementation of interfaces and a simple connection to other systems (26, 27). Also, a large number of available implementation libraries and many available examples facilitate the work of the software developer. The data specifications are widely open and can be used free of charge without restrictions (26, 27).

Eventually a proof-of-concept was developed, presented, and agreed upon by all participants. The project was registered with the local ethics committee and data protection regulations needed to be applied. The new tumor documentation system was approved by the ethics committee of the Medical faculty Heidelberg of the University of Heidelberg (vote S-287/2022) and approved by the chief privacy officer of the University Hospital Heidelberg. Also, governance structures needed to be implemented defining specific roles and allocating responsibilities.

### 2.2 Developing the prototype

In the next phase, SAP SE together with a team of documentation staff and users conducted an initial evaluation and ideation phase (a so-called sprint-0). During this intensive, six-week collaboration, a multidisciplinary team (technical experts, software designers, end users, product owners, design thinking coaches and business experts) is conducting a series of workshops. The goal was to first get an understanding of the existing challenges and requirement, subsequently identifying end user groups and their relation to each other. In the next step understanding the current as-is-process and end user needs, sore points and challenges were identified. Ultimately these efforts resulted in a strategy to define the data-processing pipeline for the future. These ideas for solution were visualized and discussed.

This procedure highlights the importance of the Design Thinking process to align needs and technical possibilities of the parties involved. Finally, an initial product backlog including a high-fidelity prototype was the tangible outcome of this sprint-0 phase.

### 2.3 Implementation, data migration and “go live”

At the beginning of the implementation phase, SAP SE and the University Hospital Heidelberg formed an interdisciplinary project team consisting of subject matter experts from the University Hospital Heidelberg (documentation staff, medical scientists, researchers, IT service staff) and SAP SE engineering. Based on the functional and non-functional requirements and insights gained during the sprint-0 phase, the newly formed project team iteratively developed a data model for the new solution based on the jointly created user stories, which documented all the different requirements and aspects. In particular, this included support for the zero-loss data migration and conversion from the existing InterSystemsCache®/KRAZTUR database.

The development process was divided into eight different sprints, with each sprint containing specific sub steps of the development process (e.g., sprint 1—basic work on the data model and visual design, step 4—developing means of registering patients with different tumor entities, tumor treatment or sprint 7—test version of the migration of data from the existing InterSystemsCache®/KRAZTUR database into DATA 5.0).

Another aspect was the extensibility and maintainability of the future data warehouse. This also included establishing a connection with the current hospital's IT infrastructure. Additional key requirements were the catalog-based configuration of data (e.g., ICD-codes, scales, gene data), search and filter capabilities of the complete data set, as well as technical support for relational and analytical data queries.

This also required a reverse engineering approach for the existing InterSystemsCache®/KRAZTUR database, to understand and map the existing data entity relations, as well as regular analysis and discussions to identify potential areas for data reorganization, data mapping and data clean-up.

In parallel, based on the initial prototype, the user interface of the new solution's different applications was designed, further refined, and implemented by the team's user experience designers and developers, continuously involving the representatives of the different future end user groups (documentation staff, medical scientists, researchers).

To ensure complete and correct data migration, representative datasets were continuously tested for data integrity, completeness, and interrogability. This was done by running both systems in parallel during the whole development process, starting from the proof-of-concept stage. First, a test phase was completed with a test system and anonymous test data. After the resolution of any impediments during the test phase, all data from the old system was transferred to the final version of DATA 5.0. Again, data sets were tested on both systems and results were compared to ensure a complete data transfer.

Complete data migration was one of the major concerns throughout the entire process. This was also one of the main reasons for a custom-made solution, as other common software solutions did not allow for a complete data transfer. By continuously reviewing the data migration process, it was ensured that no data was missing or corrupted.

Working in an iterative, sprint-based development mode (a total of eight sprints) allowed the team to showcase and review the developed increments on a regular basis to all relevant stakeholders which made it possible to consider and incorporate their feedback as early as possible.

## 3 Results

### 3.1 Technical aspects

**Figure 1:**
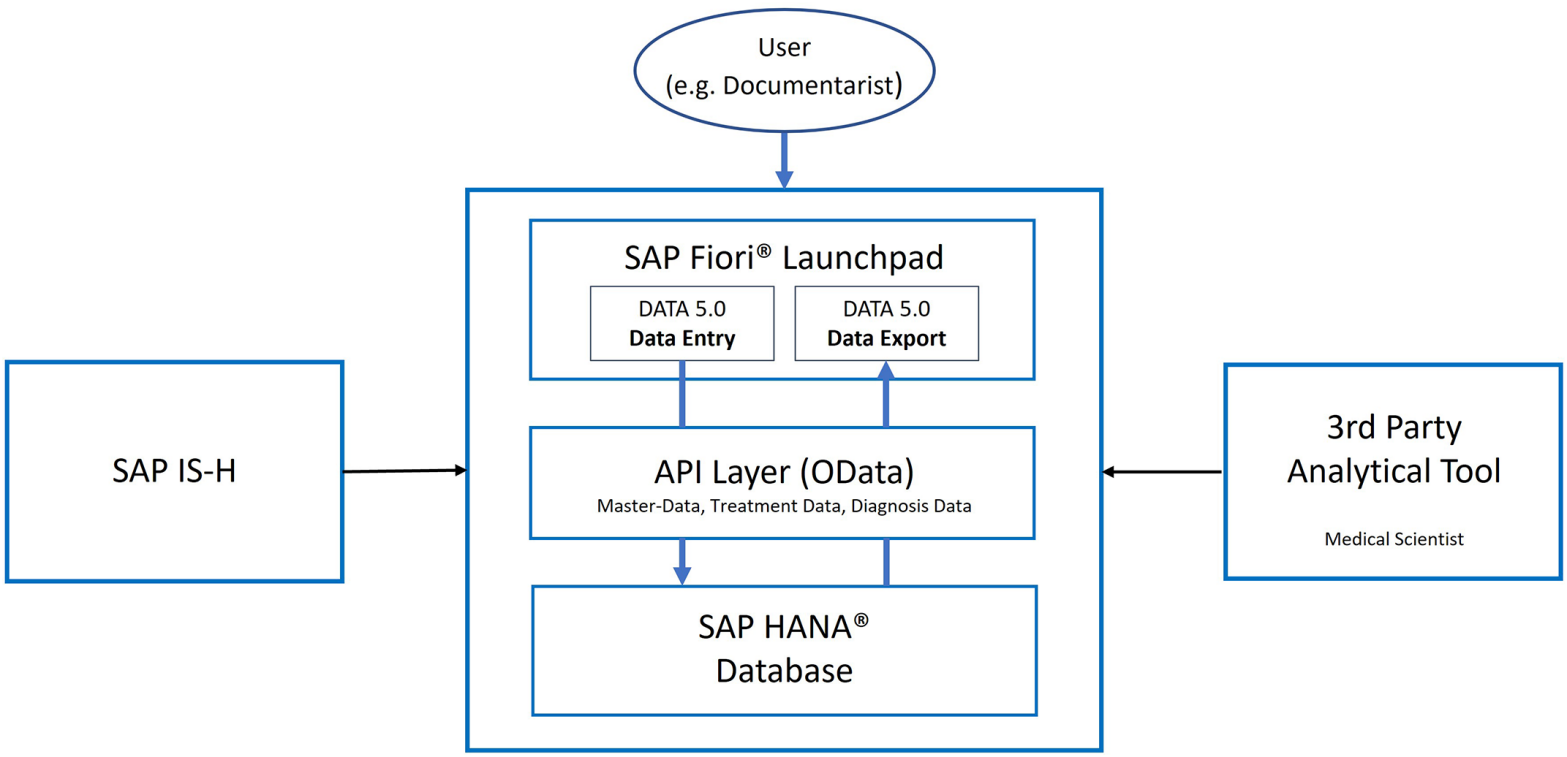
High-level Architecture. SAP HANA (+registered Trademark sign) for data storage, API (Application Programming Interfaces) Layer for data access and SAP Fiori (+registered trademark sign) Launchpad for accessing the custom applications. An optional connection to the clinic's SAP IS-H (Information System—Hospital) Health system can be leveraged for automatic master data adjustments. Third party tools can be connected for advanced analysis. OData, open data protocol.

The process described in the previous section resulted in an intuitive solution, which allows a comprehensive documentation approach, including the collection of clinical data and survival data. A safe and complete migration of the existing dataset was achieved. At the time of writing this manuscript (October 2023), DATA 5.0 includes data from 15,345 patients, with 6.7 million data points and 148,028 data registration events. The new data warehouse allows the storage and retrieval of already existing data as well as new data in a manner that allows state-of-the-art analytics. An overview of the final architecture of the data warehouse created is shown in Figure 1.

**Figure 2:**
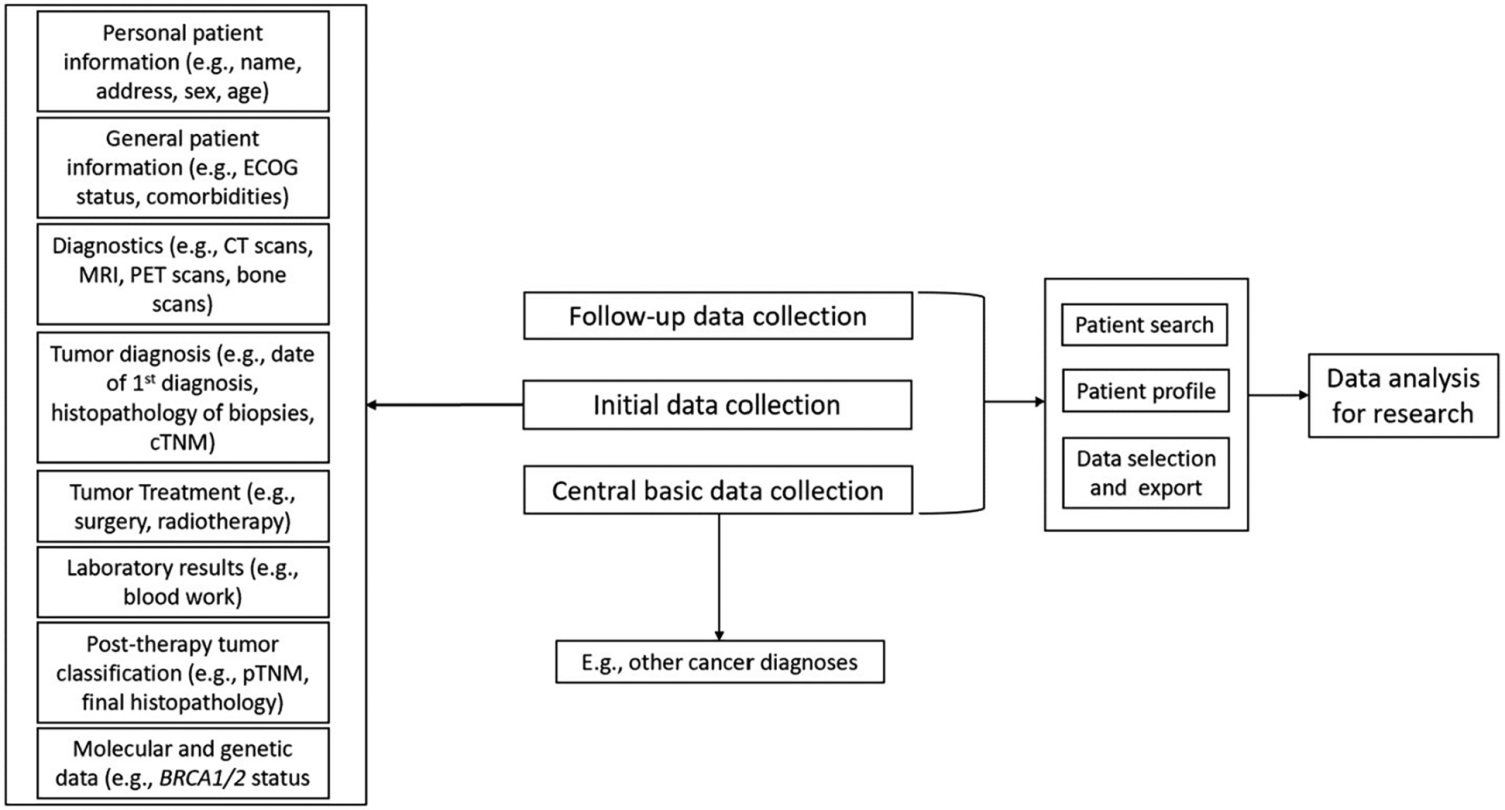
Overview of the main components. DATA 5.0 is fueled by three main data sources. These include the initial data collection during in-patient treatment, follow-up data collection throughout lifetime and the central basic data collection, which may include information on other cancer diagnoses, which are not included in DATA 5.0. Besides data collection and storage, DATA 5.0 allows data selection and individual patient search for data analysis and research.

The solution provides eight specific user-role templates, nine tumor-specific data collection templates, ten dedicated applications, over 50 tables/entities, over 190 configurable catalogs, over 400 evaluable fields (easy-to-consume by end-users), over 14,300 patients in the productive system and over 131,000 data collection logs (initial data collection, follow-up data collection, basic data collection without follow-up) (Figure 2).

**Figure 3:**
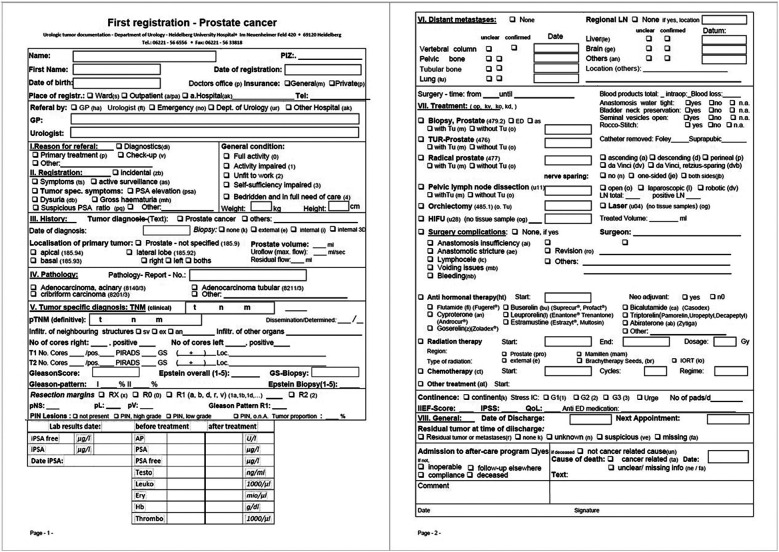
Prostate cancer—initial data registration. During the initial data registration a variety of information is entered into DATA 5.0. Prior to DATA 5.0 this was done using the above form which included information on the initial diagnosis, staging, any treatments and surgeries, pathological results, laboratory results and complications.

Each data collection includes a wide range of information, which are registered into DATA 5.0 for each follow-up visit. An example for the initial data registration for patients with prostate cancer is given in Figure 3. Data points registered during the initial data registration in the case of prostate cancer include, for example, date of the initial diagnosis, result of the initial histological examination, localization of the tumor within the prostate, staging examinations, laboratory results pre and post surgery, final TNM stage and the treatments (e.g., surgery, additional radiation therapy) the patient received.

**Figure 4:**
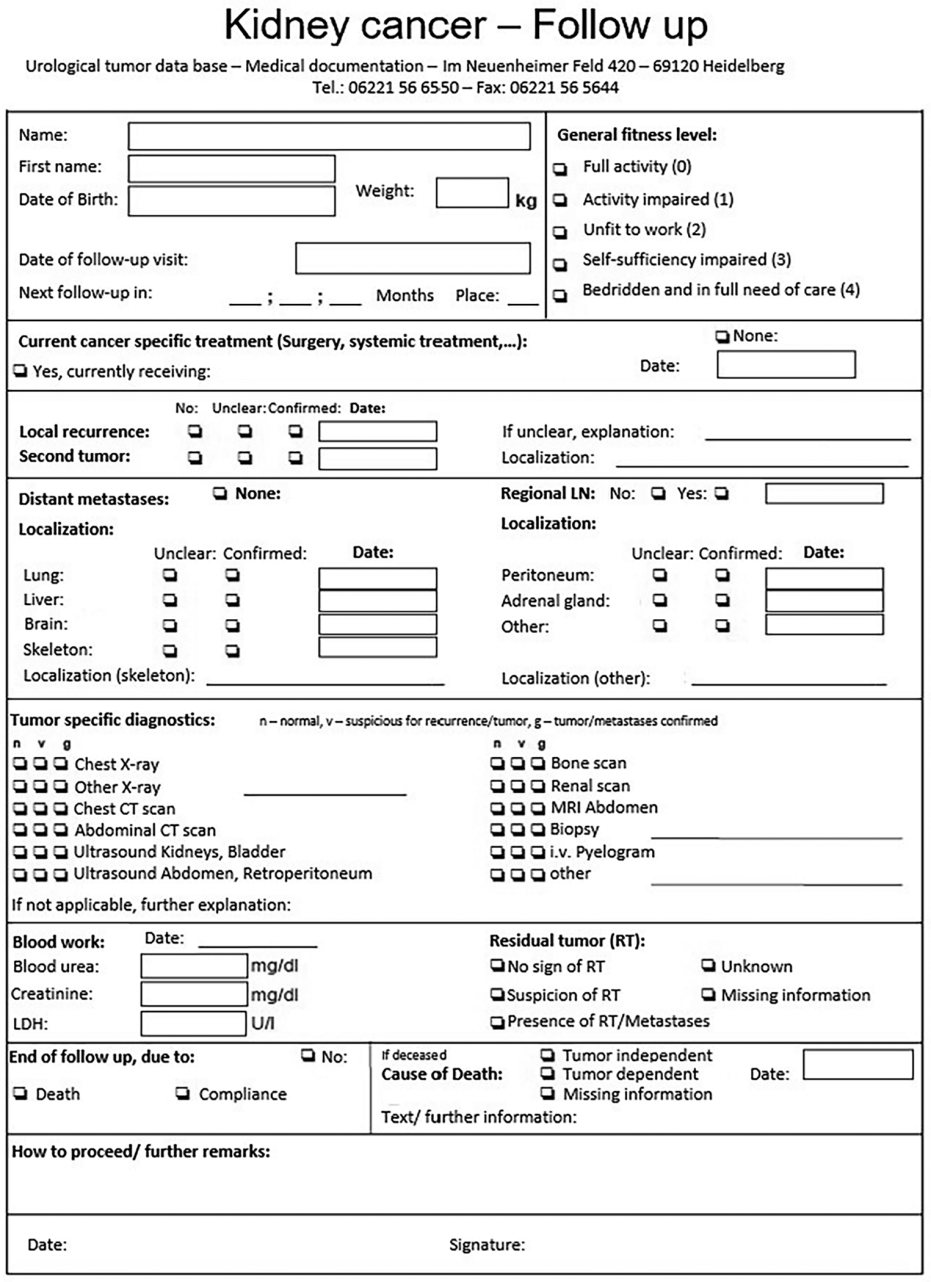
Renal cell carcinoma—follow-up questionnaire. This is an example of a questionnaire send to patients with renal cell carcinoma to gather follow-up information including information on the patients general activity level, if the patient is currently treated for renal cell carcinoma, if there are any metastases and information on further diagnostics including imaging and laboratory results.

Similar, but less detailed follow-up questionnaires are sent to the patient and their treating physician to be filled out. An example for a follow-up questionnaire for a patient treated for renal cancer is given in Figure 4. All information gathered during the initial treatment and follow-up are registered into DATA 5.0. During each data registration event the data available for each patient is evaluated regarding comprehensiveness and plausibility.

**Figure 5:**
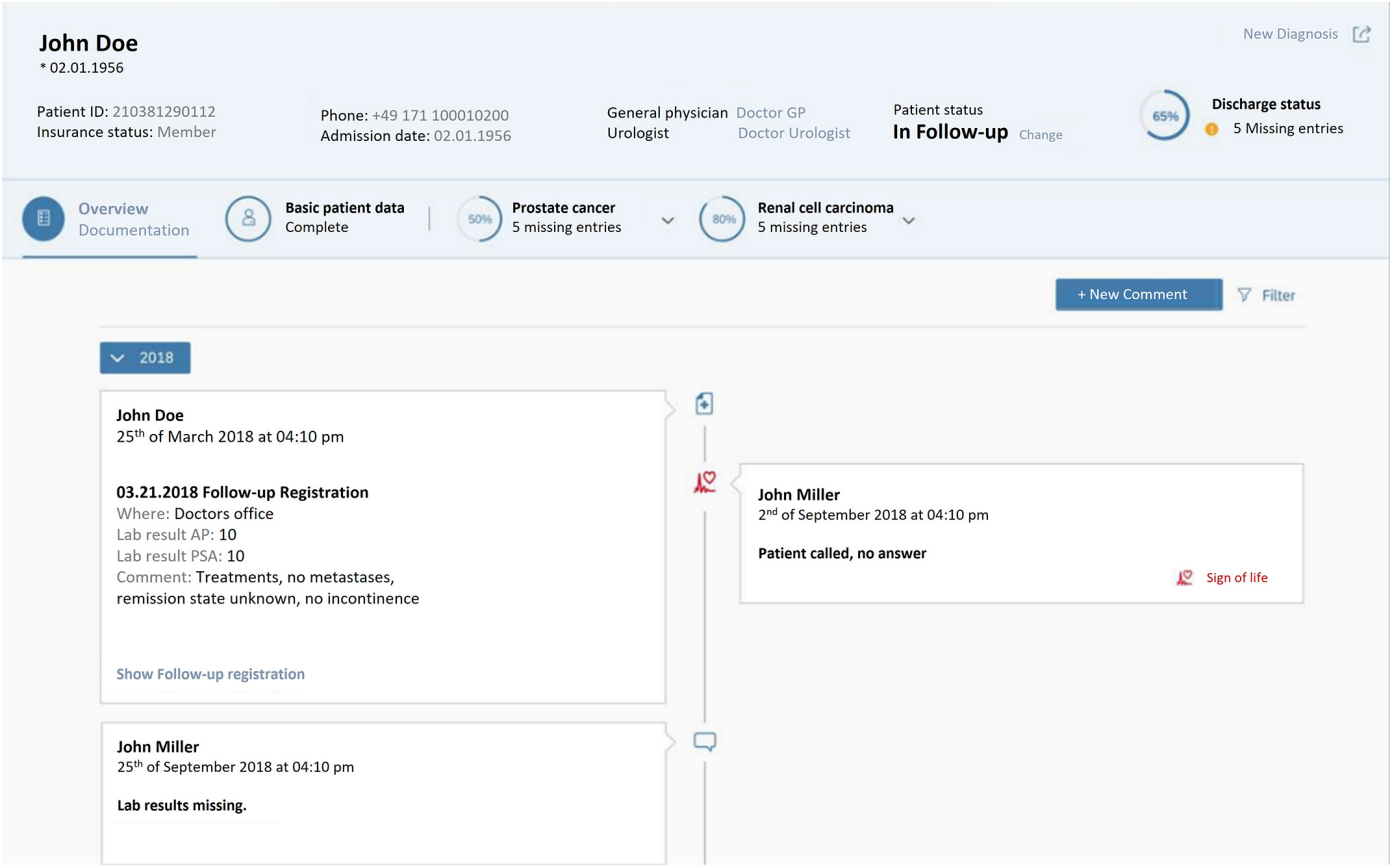
DATA 5.0 design overview—showing a typical patient file with basic information and different functional fields, including basic patient information (e.g., date of birth and contact information), fields for further information of the patient's tumor diagnosis (in this case prostate and renal cancer).

Information on each patient registered in DATA 5.0 can be accessed via a user-friendly search mask. With different sub-categories the data available is structured, can be added to and retrieved. The patient overview also helps documentation staff to navigate between different sub-categories. The patient overview also helps to assess, whether the information registration is completed, thus aiding quality control. An example of a patient overview is given in Figure 5.

One key benefit of the new solution is the user-friendly possibility to create and share basic research queries and correlations on the solution's stored data. Medical staff and researchers can work with the solution without the need of any special technical or IT-related knowledge. Queries can be handled and carried out via a simple, intuitive interface without a special training. Previously each query required a specific code to be written by especially trained staff. This gives the medical scientists and researchers a flexibility during their work and reduces the workload of the documentalist who had to support the users in the past for any kind of research-related data analysis. Data access can be limited to read-only access, to avoid any impairment with the data set.

### 3.2 Data security

In medical research, documentalists and scientists work with highly sensitive data. This becomes even more important in times of security breaches to hospital IT-infrastructures that have been reported over the past years. Therefore data security was another major concern in developing DATA 5.0. Different measures were taken to ensure the safety of the patient data stored. DATA 5.0 is hosted on a private cloud which is certified according to international standards for internet security (ISO 27001). Also, the security of this cloud is in accordance with standards from the Federal Office for Information Security and complies with its Cloud Computing Compliance Criteria Catalogue (BSI C5). These two standards are mandated by German law to allow medical data to be stored in cloud solutions. Besides these prerequisites the data itself is stored in the cloud in an encrypted form. Also, DATA 5.0 can only be accessed from certain computers and only by the documentalists, medical doctors and scientists who have been granted access to the database. Furthermore, users are only granted access to the database in their specific roles i.e., not all users have full access to the database or a right to make changes to the data set. In addition, any person who accesses the database can be tracked in real time.

### 3.3 Exemplary studies enabled by InterSystemsCache®/KRAZTUR/DATA 5.0 together with the NCT Biobank

Data from InterSystemsCache®/KRAZTUR and DATA 5.0 has been the basis for various studies and publications in the past. These highlight the valuable connection of clinical and follow-up data together with information derived from tumor tissue analysis. An example is the analysis of intratumoral heterogeneity in patients with clear cell renal carcinoma caused by the formation spatial niches in context of oncological outcome (15). A long-term survival follow-up of patients from this cohort has recently been published in which follow-up data has been analyzed in conjunction with an analysis of tumor infiltrating lymphocytes in tissue samples from the primary tumor (17).

Another example for the use of tissue samples together with clinical information is an analysis of five patients undergoing surgery for renal cell carcinoma with the formation of a venous tumor thrombus showing a mutational heterogeneity between the primary tumor and the tumor thrombus (28).

A recent project (CLINIC 5.1) by the Department of Urology of Heidelberg University Hospital focuses on establishing new forms of AI-based decision support for prostate cancer. To enable this project data from prostate cancer patients registered in DATA 5.0 has been used.

These examples highlight possible future applications and research areas for the use of clinical data together with information derived from tissue analysis as AI-decision support becomes part of the clinical routine in the treatment of cancer patients. For future applications further data sources, for example molecular analysis or imaging data, can also be added into DATA 5.0.

## 4 Discussion

At the Department of Urology at Heidelberg University Hospital, prospective data collection of general, treatment and follow-up data of urological cancer patients began in the early 1990s. To store the data, a software developed in the 1980s for the Cancer Center Heidelberg/Mannheim was utilized (1, 22). However, after more than two decades the database was outdated, and a new solution for future demands, including the option for big data analysis, was needed, while preserving all existing data. This included approx. 14,200 patients with approx. 3.6 million data points by 2022. Other requirements were, that the database would be flexible, non-proprietary, extendable for the addition of new data sources and third-party analytical tools.

Together with SAP SE, a team of medical researchers, documentation staff as well as hospital IT service began working on the development of a web-based data warehouse. This included an intensive process of understanding current and future needs, designing a user-friendly and accessible user-interface, and, most importantly, transferring all data from the old to the new database. Eventually, the new data warehouse DATA 5.0 was developed with all previously collected data being successfully preserved. DATA 5.0 allows the collection of basic patient data, information on the patient's treatment, and follow-up after discharge from the hospital including long-term follow-up by an after-care program. Additionally, DATA 5.0 also allows the addition of pathological, genetic, molecular, and imaging data. From these vast amounts of data, ultimately big data analyses and the development of a patient's digital twin should be possible.

A famous expression by data scientist Clive Humby from 2016 claims that “Data is the new oil” by which he refers to the idea that data, just like crude oil, has to be refined and processed to be valuable (29). This quote highlights how data have become a valuable economic source, just like crude oil has been for decades. But also in academics and medicine high-quality data is essential for drawing the correct conclusions and driving innovation.

Medical data is the basis for advances in cancer treatment, biomarker development and quality control, especially in a surgical specialty, or decision support. Data derived from the diagnosis, treatment and follow-up of cancer patients is especially valuable when combined with tissue and liquid biobanks (1). The connection of tissue biobanks with clinical data has already been widely recognized. Time magazine described the establishment of biobanks as one of the most important ideas changing the world in 2009 (30). There are many national and international projects. A prominent example for the connection of clinical data and tissue from biobanks on a national has been set by Sweden. An infrastructure on handling samples and corresponding information between regional and university hospitals has been established (31). Due to Sweden's uniform healthcare system, infrastructure for research and standardized data registers within the Swedish healthcare system (e.g., Prescribed Drugs Registry, the Swedish Cancer and the Cause of Death Registry) quality and patient safety can be assured (31). On an international level, BBMRI-ERIC (Biobanking and BioMolecular Resources Research Infrastructure-European Research Infrastructure Consortium), a project by the European Union, is an example for providing an infrastructure for biobanking to aid biomedical research (5). This project brings together biobanks from different European countries including researchers as well as industry partners. BBMRI-ERIC offers support for its participants regarding quality management, support with legal and ethical issues, software solutions and online tools. Eventually, this project aids the development of new treatments (32) and provides an efficient research environment. Other examples where data warehouses are utilized in the collection of data from cancer patients are a comprehensive clinical data warehouse at Rutgers Cancer Institute in New Jersey, USA, which also incorporates data from medical records, clinical trials management systems, tumor registries, biospecimen repositories, radiology and pathological archives and next generation sequencing services (33). This database, described in 2017 based on a thousand cases, was developed to support precision medicine applications in the treatment of oncological patients (33). In contrast, DATA 5.0 incorporates data from over 15,000 patients. In addition, at the University Hospital Heidelberg tissue samples are stored in the NCT-Biobank as part of the BioMaterialBank Heidelberg which was established in 2011 (34). This allows information derived from DATA 5.0 to be connected to results from pathological and molecular analyses. DATA 5.0 as well as the previous database have been the basis for research projects published by our group which allows results e.g., from tissue analysis to be put into a clinical context (15, 17, 35–37).

A crucial aspect of the previous tumor documentation system and now DATA 5.0 is the structured after-care program (1) allowing long-term outcome analysis without having to set up a trial for answering a specific research question (15, 16). This is especially important in diseases with long survival rates such as prostate cancer with a 5-year survival rate of 98% combined over all stages (38).

In the treatment of cancer patients quality control is of utmost importance. By constantly evaluating the data collected during the treatment of cancer patients adjustments can be made and improve the quality of a specific treatment (e.g., a surgical approach) (39). This idea of analyzing patient data regarding treatment outcome and making adjustments where needed to improve a certain treatment has been described as “a learning health system” (39). By analyzing data on different surgical techniques DATA 5.0 can help to answer the question which surgical approach might be best for a specific patient group. An example is the analysis of salvage prostatectomy results, which showed that even in a salvage setting, robot assisted, retzius-sparing radical prostatectomy is feasible (16).

In order to draw conclusions about the optimal therapy for individual patients, it is essential to evaluate big data that has already been collected as well as big data that will be collected in the future in order to determine the relevance and impact of individual patient and tumor information with regard to diagnosis, therapy decisions and prognosis. To do this, the appropriate infrastructure and tools for comprehensive and large-scale data analyses must be available. This includes long-term documentation of patient outcomes and therapy successes on the one hand and data mining tools for extracting therapy-relevant information on the other. Especially long-term data collection is crucial.

Innovative approaches for the development of new predictive and prognostic parameters, particularly in multimodal therapy, based on the existing large amounts of data with high-quality clinical information, are urgently required. The development of a data warehouse that registers and stores the diverse results from diagnostics and therapy—where possible in real time is a prerequisite. As a high-quality bidirectional open and expandable hub, DATA 5.0 allows the multivariate analysis of e.g., imaging, histological, molecular biological and surgical data with all marketable analysis programs. DATA 5.0 explicitly allows interaction with other software such as Onkostar, i.s.h.med, RaySearch, SPSS, Qlucore etc. This can also aid collaboration with other research institutions allowing the exchange of information and data for specific research projects. For these special data protection regulations must be applied, e.g., only allowing information to be exchanged in an anonymous form to ensure data protection laws and patient privacy.

Long-term data on patient outcomes are essential for evaluating the effectiveness and impact of new forms of artificial intelligence-based decision support. Well-curated data in medicine as the basis for convolutional neural networks and artificial intelligence-based applications is leading to a new level of precision medicine, aiming at personalized medicine in terms of diagnosis and therapy for individual patients. This individualization will be facilitated by the development of “digital twin” technology. The CLINIC 5.1 project, funded by the German Federal Ministry of Economics and Technology and led by the Department of Urology at the University Hospital of Heidelberg, has established new forms of AI-based decision support for prostate cancer. Existing patient data in DATA 5.0 enabled the creation of innovative decision support tools based on previous therapies, diagnoses and studies in all phases of diagnosis, therapy recommendation and therapy implementation—a step towards personalized medicine with the possibility of individually tailored therapy recommendations. The technology of the “digital twin” has great potential to open up new perspectives for simulating and performing more precise diagnostics and therapies. Another application for DATA 5.0 could be the use of real-world data for precision oncology (14) and other aspects such as evaluation of treatment regimes, technical innovations or even health care policy (40).

The growing application of AI in healthcare, including advanced machine learning and Large Language Models (LLM), introduces new ethical challenges. Although our data has historically been used for basic and clinical research, the rise of AI underscores the importance of clarifying consent, addressing algorithmic biases, and ensuring that patient information is used responsibly. A recurring ethical concern, especially for databases established decades ago, is how previous patient consent aligns with modern research methodologies that were unforeseen at the time of data collection. Traditional consent forms did not specifically anticipate applications such as AI-driven analytics or LLM training.

It is therefore critical to amend consent forms to include AI applications for prospective data collection. The use of already existing data sets for AI applications creates a dilemma that until now remains largely unresolved. The most straight forward and ethically correct approach would be to ask for the patient's consent for use of their data for AI. This strategy is in alignment with current regulatory statutes such as the General Data Protection Regulation (GDPR) of the European Union which explicitly permits patients to withdraw their consent at any point of time with deletion of their data from any data bases. If, in the future, already existing data sets should become available for AI applications a national and international framework to enable the use of these data will be mandatory.

Limitation to DATA 5.0 include, that it only focuses on data from urological cancer patients. However, with DATA 5.0 being a successful prototype for a new web-based approach to organizing and storing data from cancer patients for research purposes, DATA 5.0 could be utilized for the collection of data for other tumor entities as well as benign conditions. As DATA 5.0 is web-based, it is not limited to specific computer systems. The project DATA 5.0 also highlights the successful cooperation of an academic institution and an industry partner. By joining forces from academia and industry new, customer specific solutions can be developed. DATA 5.0 is a lighthouse project highlighting this special cooperation.

To fully take advantage of DATA 5.0 some aspects still need to be established. To allow for an easier and quicker transfer of data into DATA 5.0 a direct link to the hospital's clinical information system should be established. This could also improve data quality as error from manual data transfer can be avoided. Another aspect is data retrieval for medical research. If data could be directly retrieved in a specific format that is needed for analytical tools the process of data selection and analysis could be more efficient and thus expedite critical research projects.

## Conclusion

This project emphasizes the importance of long-term data collection for oncological research and patient care. DATA 5.0 also highlights the sustainability and evolution of long-term data collection and curation. Data have become one of the most important aspects in every aspect of human life. Especially in health care and cancer treatment data are the basis for every advancement made. DATA 5.0 aims to not only allow long-term, longitudinal data collection of patients with urological cancers but also aids translational research, big-data analysis and eventually AI-applications to improve patient care.

## Data Availability

The original contributions presented in the study are included in the article/Supplementary Material, further inquiries can be directed to the corresponding author.
